# Associations of Social Support, Loneliness, and Physical Activity with Cognitive Function in Older Adults with Mild Cognitive Impairment

**DOI:** 10.3390/jcm15187207

**Published:** 2026-09-17

**Authors:** Juan Miguel Muñoz-Perete, Yolanda Castellote-Caballero, María del Carmen Carcelén-Fraile, José Juan Castro-Sánchez

**Affiliations:** 1 Department of Health Sciences, Faculty of Health Sciences, University of Jaén, 23071 Jaen, Spain; jmmunoz@ujaen.es (J.M.M.-P.);; 2 Department of Health Sciences, Faculty of Health Sciences, University of Atlántico Medio, 35017 Las Palmas de Gran Canaria, Spain; 3 Department of Psychology, Sociology and Social Work, University of Las Palmas de Gran Canaria, 35001 Las Palmas de Gran Canaria, Spain; josejuan.castro@ulpgc.es

**Keywords:** mild cognitive impairment, physical activity, social support, loneliness, executive function, attention

## Abstract

**Objectives:** This study investigated how perceived social support, loneliness, and physical activity are related to cognitive performance among older adults with mild cognitive impairment. **Methods:** This cross-sectional investigation included 112 community-dwelling older adults with mild cognitive impairment. Perceived social support, loneliness, and physical activity were evaluated with the Multidimensional Scale of Perceived Social Support (MSPSS), UCLA Loneliness Scale, and International Physical Activity Questionnaire (IPAQ), respectively. Cognitive functioning was characterized through a battery comprising the Montreal Cognitive Assessment (MoCA), Isaac Set Test, Trail Making Test Parts A and B (TMT-A and TMT-B), Digit Symbol Substitution Test (DSST), and d2 Attention Test. Relationships among the study variables were examined through correlation and multivariable linear regression analyses, with age, sex, and educational level included as covariates. To address multiplicity, Benjamini–Hochberg false discovery rate (FDR) adjustment was conducted independently for the predefined families of correlations, main-effect regression coefficients, and interaction terms. **Results:** Significant bivariate relationships with cognitive performance were observed for perceived social support, loneliness, and physical activity, with all 18 correlations retaining significance following FDR adjustment. After covariate adjustment, 12 of the 36 main-effect coefficients met the FDR-corrected significance threshold. Among the factors examined, physical activity displayed the broadest pattern of independent associations, encompassing global cognition, visual attention and processing speed, executive function, processing speed, and selective attention and concentration. Perceived social support was independently related to global cognition, visual attention and processing speed, and executive function. In contrast, five effects that reached nominal significance did not withstand FDR adjustment; these included physical activity in relation to verbal fluency and loneliness in relation to global cognition and TMT-B performance. Adding the interaction terms increased the variance explained by the models for MoCA (ΔR^2^ = 0.054, *p* = 0.020) and TMT-B (ΔR^2^ = 0.059, *p* = 0.010); however, no individual interaction term met the FDR-corrected significance threshold. **Conclusions:** Among the factors examined, physical activity exhibited the broadest pattern of independent relationships with cognitive performance in older adults with MCI, although the association with verbal fluency was no longer significant after adjustment for multiple testing. Perceived social support was related to a more limited set of cognitive outcomes, while associations involving loneliness were attenuated after FDR adjustment. Because no individual interaction term reached FDR-corrected significance, the interaction results should be regarded as exploratory. The cross-sectional nature of the study precludes conclusions about causality or protective effects. Future longitudinal research incorporating objective assessments of physical activity is warranted to establish the temporal direction and clinical significance of these associations.

## 1. Introduction

The progressive aging of the global population has important implications for cognitive health. World Health Organization (WHO) projections indicate that the population aged 60 years and older will exceed 2 billion by 2050, approximately twice the current number [1]. Longer life expectancy, while reflecting improvements in healthcare and living standards, also increases the number of individuals exposed to age-related cognitive decline and neurodegenerative conditions [2]. Within this context, mild cognitive impairment (MCI) is particularly relevant because it represents a clinical state between the cognitive changes associated with normal aging and dementia [3]. Individuals with MCI present measurable cognitive deficits beyond those expected for their age and educational background, but generally retain independence in everyday functioning. Importantly, this population faces a substantially greater likelihood of progressing to dementia than cognitively healthy older adults [4].

The growing prevalence of MCI has generated considerable scientific interest in identifying modifiable factors that may influence cognitive trajectories during aging [5]. Although age and genetic predisposition are well-established determinants of cognitive decline, increasing evidence suggests that psychosocial and lifestyle-related factors play a crucial role in maintaining cognitive health and delaying the progression of cognitive impairment [6]. In particular, perceived social support, loneliness, and physical activity have emerged as potentially modifiable determinants of cognitive function in older adults [7].

Social support encompasses several forms of interpersonal resources, including emotional, instrumental, informational, and appraisal support [8]. Its relevance in later life extends beyond general well-being, as greater social support has been linked to better quality of life, fewer depressive symptoms, greater resilience, and lower mortality. Evidence also points to a relationship between perceived social support and cognitive functioning, with higher levels of support being associated with better performance in areas such as memory, executive function, language, and attention [9]. Longitudinal research further suggests that maintaining strong social relationships may be related to greater cognitive reserve, potentially supporting cognitive functioning in the presence of age-related neuropathological changes [10].

Several pathways may underlie the relationship between social support and cognitive functioning. Interacting with others regularly engages communication, memory retrieval, problem-solving, and emotional regulation, providing repeated opportunities for cognitive activation [11]. Social support may also be related to lower physiological stress burden by limiting prolonged activation of stress-response systems, including the hypothalamic–pituitary–adrenal axis, with potential implications for cortisol exposure and neuroinflammatory processes involved in neurodegeneration. In contrast, loneliness represents another psychosocial factor that has been linked to cognitive decline in later life [12]. Unlike social isolation, which reflects the objective extent of social contact, loneliness refers to the subjective experience of perceiving one’s social relationships as insufficient or unsatisfactory [13]. Persistent feelings of loneliness in older adults have been associated with a faster decline in cognitive abilities and a greater likelihood of developing dementia [14]. Several interconnected pathways have been proposed to account for these relationships. Loneliness has been related to greater psychological stress, altered hypothalamic–pituitary–adrenal axis activity, systemic inflammatory processes, sleep disturbances, and lower participation in cognitively stimulating activities [15]. Collectively, these factors may be associated with structural and functional alterations in brain regions involved in processes such as memory and executive control [16].

Physical activity constitutes another key modifiable determinant of cognitive health. Extensive evidence from epidemiological, clinical, and experimental studies indicates that regular physical activity is associated with improved cognitive performance and reduced risk of cognitive decline [17]. The protective effects of physical activity appear to involve multiple physiological pathways, including enhanced cerebral blood flow, increased neurogenesis, improved synaptic plasticity, reduced neuroinflammation, greater metabolic regulation, and increased production of neurotrophic factors such as brain-derived neurotrophic factor (BDNF) [18]. These mechanisms support neuronal survival and facilitate functional connectivity in brain regions vulnerable to aging-related degeneration [19].

Importantly, physical activity has been linked to improvements across diverse cognitive domains [20]. Aerobic and moderate-to-vigorous physical activities have demonstrated beneficial effects on global cognition, executive functions, attention, processing speed, and memory. Among individuals with MCI, physically active lifestyles have been associated with slower cognitive decline and lower rates of progression to dementia [21]. Nevertheless, despite the substantial body of evidence supporting the cognitive benefits of physical activity, findings remain heterogeneous, and the magnitude of these associations may vary according to activity intensity, duration, and individual characteristics [22].

Despite growing evidence supporting the independent roles of social support, loneliness, and physical activity in cognitive aging, relatively few studies have examined the combined and independent associations of these modifiable factors with specific cognitive domains in older adults with MCI. Although memory impairment has traditionally been considered the hallmark feature of MCI, growing evidence indicates that deficits frequently extend to other cognitive domains, including verbal fluency, executive functioning, processing speed, attention, and concentration [23]. Impairments in these domains have been associated with reduced daily functioning, lower quality of life, and an increased risk of progression to dementia [24]. In particular, executive dysfunction and reduced processing speed have been identified as early markers of neurodegenerative processes, whereas deficits in verbal fluency and attentional performance may reflect widespread alterations in neural networks involved in cognitive control and information processing [25]. Consequently, examining multiple cognitive domains provides a more comprehensive understanding of cognitive vulnerability in older adults with MCI and may help identify factors associated with cognitive preservation or decline [26].

Addressing these gaps is particularly relevant given the increasing emphasis on multidomain approaches to dementia prevention and healthy cognitive aging [27]. Understanding how psychosocial resources and lifestyle behaviors relate to cognitive functioning in older adults with MCI may contribute to the development of more effective preventive and therapeutic strategies aimed at preserving cognitive health and delaying disease progression [28].

Therefore, this study investigated the relationships between perceived social support, loneliness, and physical activity and cognitive functioning in older adults with mild cognitive impairment. Cognitive performance was considered both globally and across specific domains, including verbal fluency, executive function, processing speed, and attention. On the basis of previous evidence, we hypothesized that greater perceived social support and higher physical activity levels would be related to better cognitive performance, whereas greater loneliness would be related to poorer performance. We further hypothesized that these associations would persist after adjustment for relevant demographic covariates.

## 2. Materials and Methods

### 2.1. Study Design

The study followed a cross-sectional observational design to characterize the variables of interest within the study population. Data were collected from May to July 2026. All study procedures were conducted in accordance with methodological standards for observational research and the ethical principles governing research involving human participants. Ethical approval was granted by the Ethics Committee of the University of Jaén (approval code: 20260414-2/ABR.PRY).

### 2.2. Participants

Participants were considered eligible for inclusion if they met all of the following criteria: (i) were aged 65 years or older; (ii) had a previously established clinical diagnosis of mild cognitive impairment (MCI), documented by a qualified healthcare professional and established according to Petersen’s criteria [29]; and (iii) provided written informed consent after receiving information about the study aims and procedures. According to these criteria, MCI was characterized by evidence of cognitive decline, objective cognitive impairment relative to age- and education-adjusted expectations, preservation of independence in functional abilities, and absence of dementia. The Montreal Cognitive Assessment (MoCA) was subsequently administered by the research team as a complementary cognitive screening measure to characterize the cognitive status of the sample and corroborate the previously established diagnosis, rather than as a stand-alone diagnostic criterion for MCI.

Individuals were excluded if they met any of the following conditions: (i) diagnosis of moderate-to-severe dementia or any other form of advanced cognitive decline; (ii) presence of severe physical or medical conditions, including significant cardiovascular or respiratory diseases, or musculoskeletal disorders; (iii) history of major psychiatric disorders, such as schizophrenia or major depressive disorder, or substance use; and (iv) inability to follow basic instructions or lack of sufficient family or social support.

### 2.3. Outcomes

Sociodemographic and clinical information was collected to describe the characteristics of the study sample. Recorded variables included age and body weight, together with marital status (married, single, separated or divorced, or widowed), employment status (retired, unemployed, or employed), and educational attainment (no formal education, primary education, secondary education, or university studies). These variables were used to characterize the study sample.

#### 2.3.1. Cognitive Impairment

The cognitive impairment was evaluated using the Montreal Cognitive Assessment (MoCA). This brief instrument comprises 12 tasks covering seven cognitive domains: visuospatial and executive abilities, naming, attention, language, abstraction, memory, and orientation. The assessment includes activities such as figure tracing, cube copying, clock drawing, reverse digit recall, sustained attention, sentence repetition, verbal fluency, abstract reasoning, and immediate and delayed recall. Scores range from 0 to 30, with scores of 26 or above generally considered indicative of normal cognitive functioning [30]. According to the MoCA interpretative framework, scores between 18 and 25 are consistent with mild cognitive impairment, whereas lower scores indicate more severe levels of cognitive impairment. In the present study, the MoCA was used as a complementary cognitive screening measure to characterize and corroborate the previously established clinical diagnosis of MCI rather than as a stand-alone diagnostic criterion.

#### 2.3.2. Verbal Fluency

Verbal fluency was evaluated with the Isaac Set Test. Participants were asked to generate as many words as possible from predefined semantic categories (animals, fruits, cities, and colors) within 60 s. Each category contributes up to 10 points, yielding a maximum total score of 40, with higher scores reflecting better verbal fluency [31].

#### 2.3.3. Executive Functions

The Trail Making Test (TMT) was administered to evaluate visual attention, processing speed, and executive function. The TMT comprises two parts. In TMT-A, participants connect numbered circles in ascending numerical order, providing a measure of visual attention and processing speed. TMT-B requires participants to alternate sequentially between numbers and letters and primarily evaluates executive function [32]. Performance is based on completion time, with longer times indicating poorer performance.

#### 2.3.4. Processing Speed

Processing speed was evaluated using the Digit Symbol Substitution Test (DSST) [33]. Participants used a reference key to pair numbers with their corresponding symbols and entered the appropriate symbol beneath each number. Performance was quantified as the total number of correct substitutions completed within 90 s, with higher scores indicating better processing speed.

#### 2.3.5. Selective Attention and Concentration

Selective attention and concentration were assessed using the d2 Attention Test [34], a widely used neuropsychological instrument for evaluating selective attention, concentration, and processing speed. The test consists of identifying and marking specific target stimuli among a large number of visually similar stimuli, requiring sustained attention and the ability to quickly discriminate relevant from irrelevant information. The test provides various performance indices, including attentional efficiency, concentration capacity, processing speed, and error control, allowing for a comprehensive evaluation of the participant’s attentional functioning.

#### 2.3.6. Social Support

Perceived social support was measured using the Multidimensional Scale of Perceived Social Support (MSPSS) [35]. This 12-item self-administered questionnaire evaluates perceived support from three sources: family, friends, and significant others. The items address emotional, instrumental, and affective dimensions of perceived support and are organized into three corresponding subscales. The MSPSS provides both an overall score and source-specific scores, with higher values reflecting greater perceived social support.

#### 2.3.7. Loneliness

Loneliness was assessed using the UCLA Loneliness Scale [36], a widely used instrument for measuring subjective loneliness and the degree of dissatisfaction with social relationships. The scale evaluates feelings related to social isolation, lack of companionship, and emotional disconnection from others, allowing for the identification of the subjective experience of loneliness regardless of the number of existing social contacts. Higher scores indicate greater levels of perceived loneliness.

#### 2.3.8. Physical Activity Level

Physical activity was assessed using the International Physical Activity Questionnaire (IPAQ) [37]. Participants reported the frequency and duration of physical activity performed over the previous seven days, including vigorous- and moderate-intensity activities and walking, as well as time spent sedentary. Responses were used to estimate energy expenditure, expressed as metabolic equivalent minutes per week (MET-min/week), and to categorize participants according to their level of physical activity.

#### 2.3.9. Confounding Variables

Age, sex, and educational level were included as covariates in the regression models due to their well-established associations with cognitive function in older adults. Age is one of the strongest determinants of cognitive performance and has been consistently associated with age-related cognitive decline. Sex was considered because previous studies have reported differences in cognitive performance, physical activity patterns, and psychosocial characteristics between men and women. Educational level was included as an indicator of cognitive reserve, as higher educational attainment has been associated with better cognitive functioning and may mitigate the clinical expression of cognitive impairment.

### 2.4. Sample Size Calculation

Sample size and statistical power were calculated using G*Power software, version 3.1.9.7. Considering the cross-sectional design and the objective of examining associations between psychosocial factors, physical activity, and cognitive function, the calculation was based on a multiple linear regression model. A 95% confidence level, an alpha error probability of 0.05, and 80% statistical power were established as reference parameters. Assuming a medium effect size, the minimum required sample size was estimated at 110 participants.

### 2.5. Data Analysis

Statistical analyses were carried out using IBM SPSS Statistics for Windows, version 22.0 (IBM Corp., Armonk, NY, USA). Sample characteristics were summarized using descriptive statistics. Continuous variables are reported as mean ± standard deviation (SD) or median and interquartile range (IQR), as appropriate, while categorical variables are presented as frequencies and percentages. Distributional assumptions for continuous variables were evaluated with the Shapiro–Wilk test together with visual examination of histograms and Q–Q plots. The cognitive outcomes considered in the analyses were global cognition (MoCA), verbal fluency (Isaac Set Test), visual attention and processing speed (TMT-A), executive function (TMT-B), processing speed (DSST), and selective attention and concentration (d2 Attention Test). Bivariate associations between psychosocial variables, physical activity, and cognitive outcomes were examined using Pearson’s correlation coefficients. Given the non-normal distributions of TMT-A and TMT-B completion times, sensitivity analyses using Spearman’s rank correlation coefficients were additionally performed for these two outcomes. These analyses yielded comparable coefficients, with the same direction and statistical significance of the associations, and did not alter the interpretation of the findings. Multiple linear regression models were subsequently fitted to determine the independent associations of perceived social support, loneliness, and physical activity with cognitive performance. A separate model was estimated for each cognitive outcome, with age, sex, and educational level entered as covariates. Potential interactive associations among perceived social support, loneliness, and physical activity were further explored using hierarchical multiple linear regression models for each cognitive outcome. Before constructing the interaction terms, the three predictors were mean-centered. The first block comprised age, sex, educational level, and the main effects of perceived social support, loneliness, and physical activity. The second block simultaneously added three pairwise interaction terms: perceived social support × loneliness, perceived social support × physical activity, and loneliness × physical activity. The contribution of this second block was assessed from the change in explained variance (ΔR^2^) and the corresponding F-change test. Interactions reaching nominal statistical significance were further explored through simple-slope analyses. Multicollinearity was evaluated using variance inflation factors (VIFs). Regression results are reported as coefficients (β), 95% confidence intervals (95% CIs), and *p*-values. To address multiple testing, the Benjamini–Hochberg false discovery rate (FDR) procedure was implemented independently within three prespecified families of comparisons: (i) 18 correlations of the MSPSS total score, loneliness, and physical activity with the six cognitive outcomes; (ii) 36 main-effect regression coefficients across the cognitive outcome models; and (iii) 18 interaction terms from the hierarchical regression models. FDR-adjusted *p*-values (q-values) were calculated separately within each family. Unadjusted *p*-values < 0.05 (two-tailed) were considered nominally significant, whereas statistical significance after correction for multiple comparisons was defined as q < 0.05. Unadjusted *p*-values and their corresponding FDR-adjusted q-values are provided in Supplementary Table S1.

## 3. Results

**Figure 1:**
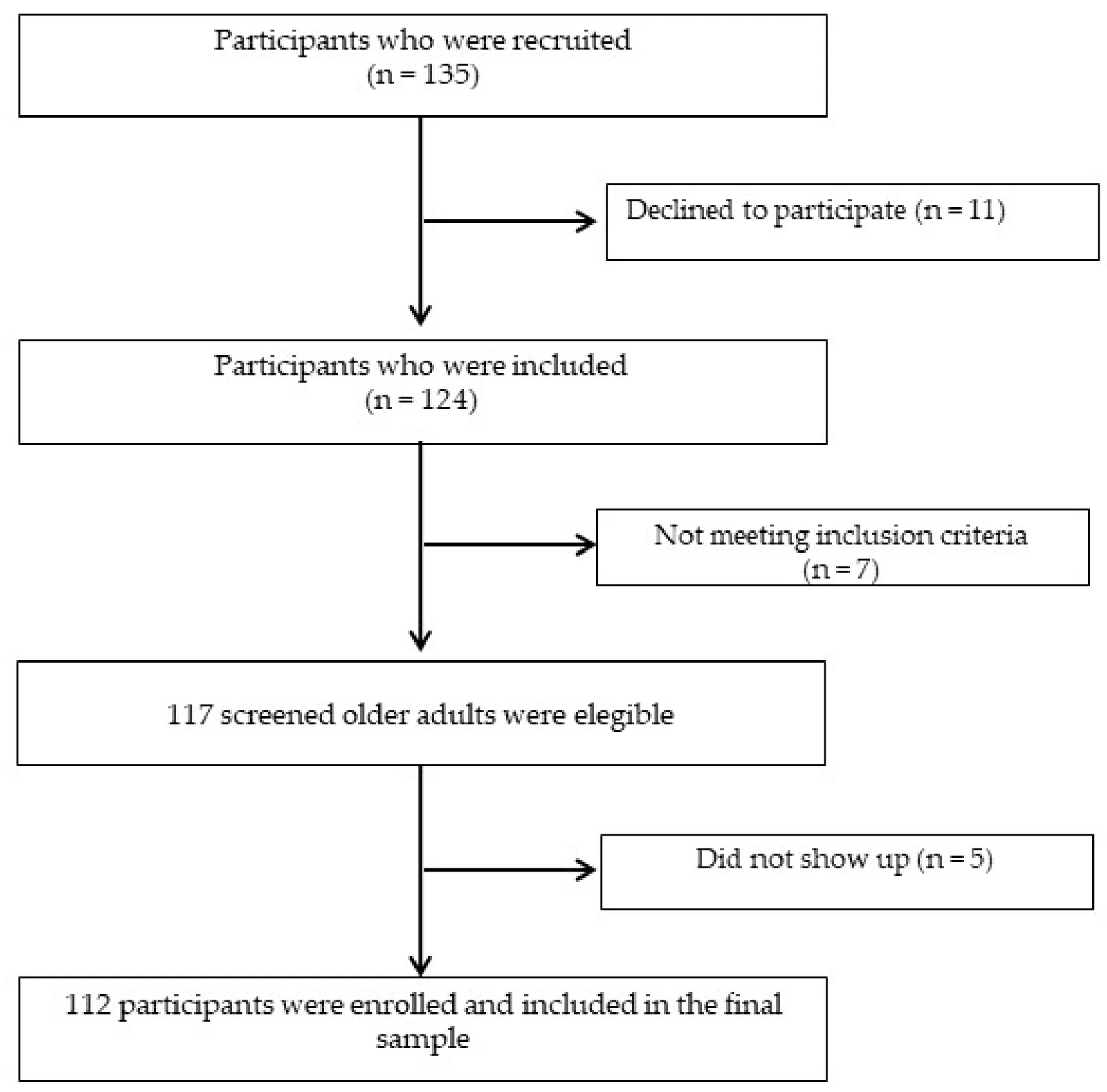
Flowchart summarizing participant recruitment, eligibility screening, and final sample inclusion.

A total of 135 older adults were initially recruited for participation in the study. Of these, 11 individuals declined to participate, resulting in 124 participants being assessed for eligibility. Subsequently, seven participants were excluded because they did not meet the inclusion criteria. Therefore, 117 older adults were considered eligible for participation. During the assessment phase, five eligible participants did not attend the evaluation sessions and were consequently excluded from the final analysis. As a result, 112 older adults with mild cognitive impairment were enrolled and included in the final study sample. The participant recruitment and selection process are summarized in Figure 1.

### 3.1. Sociodemographic Characteristics

A total of 112 older adults with mild cognitive impairment were included in the study. Participants had a mean age of 71.04 ± 3.31 years. Women represented the majority of the sample (63.4%), whereas 36.6% were men. Most participants were retired (72.3%), while 27.7% were unemployed. Regarding marital status, more than half of the participants were married (54.5%), followed by those who were divorced, separated, or widowed (27.7%) and single individuals (17.9%). In terms of educational attainment, primary education was the most common educational level (54.5%), followed by secondary education (21.4%), no formal education (17.0%), and university education (7.1%). The mean body weight was 72.88 ± 12.68 kg, mean height was 1.67 ± 0.13 m, and mean body mass index (BMI) was 26.41 ± 4.91 kg/m^2^. According to World Health Organization criteria, participants were, on average, within the overweight range. Detailed sociodemographic and anthropometric characteristics of the study population are presented in Table 1.

### 3.2. Correlations Between Psychosocial Factors, Physical Activity, and Cognitive Function

Perceived social support was positively associated with global cognitive performance. Specifically, the total MSPSS score showed a moderate positive correlation with MoCA scores (r = 0.413, *p* < 0.001). Similar associations were observed for the Family Support (r = 0.304, *p* = 0.001), Friends Support (r = 0.325, *p* < 0.001), and Significant Others Support (r = 0.290, *p* = 0.002) subscales.

Higher levels of perceived social support were also associated with better performance across several cognitive domains. The total MSPSS score correlated positively with verbal fluency (Isaac Test; r = 0.234, *p* = 0.013), processing speed (DSST; r = 0.294, *p* = 0.002), and selective attention and concentration (d2 Attention Test; r = 0.368, *p* < 0.001). Conversely, higher MSPSS scores were associated with shorter completion times on the Trail Making Test Part A (r = −0.404, *p* < 0.001) and Part B (r = −0.449, *p* < 0.001), indicating better executive functioning.

The MSPSS subscales showed a similar pattern of associations. Family Support was positively correlated with MoCA (r = 0.304, *p* = 0.001), verbal fluency (r = 0.252, *p* = 0.007), DSST (r = 0.247, *p* = 0.009), and d2 performance (r = 0.301, *p* = 0.001), and negatively correlated with TMT-A (r = −0.333, *p* < 0.001) and TMT-B (r = −0.334, *p* < 0.001). Friends Support was positively associated with MoCA (r = 0.325, *p* < 0.001), DSST (r = 0.226, *p* = 0.017), and d2 performance (r = 0.283, *p* = 0.002), and negatively associated with TMT-A (r = −0.258, *p* = 0.006) and TMT-B (r = −0.342, *p* < 0.001). Significant Others Support showed positive correlations with MoCA (r = 0.290, *p* = 0.002), verbal fluency (r = 0.225, *p* = 0.017), DSST (r = 0.218, *p* = 0.021), and d2 performance (r = 0.245, *p* = 0.009), and negative correlations with TMT-A (r = −0.316, *p* = 0.001) and TMT-B (r = −0.326, *p* < 0.001).

Loneliness was negatively associated with cognitive performance. Higher UCLA Loneliness Scale scores were correlated with lower MoCA scores (r = −0.358, *p* < 0.001), poorer verbal fluency (r = −0.198, *p* = 0.036), lower processing speed (r = −0.270, *p* = 0.004), and reduced attentional performance (r = −0.314, *p* = 0.001). Furthermore, greater loneliness was associated with longer completion times on the TMT-A (r = 0.312, *p* = 0.001) and TMT-B (r = 0.365, *p* < 0.001).

Physical activity level was consistently associated with cognitive outcomes. Higher IPAQ scores were positively correlated with global cognitive performance (MoCA: r = 0.428, *p* < 0.001), verbal fluency (Isaac Test: r = 0.262, *p* = 0.005), processing speed (DSST: r = 0.339, *p* < 0.001), and attentional performance (d2 Attention Test: r = 0.428, *p* < 0.001). In addition, greater physical activity was associated with shorter completion times on the TMT-A (r = −0.400, *p* < 0.001) and TMT-B (r = −0.452, *p* < 0.001), indicating better executive functioning.

Overall, perceived social support demonstrated moderate positive associations with cognitive performance, whereas loneliness showed consistent negative associations across all cognitive measures. Physical activity exhibited the strongest and most consistent associations, being significantly related to all cognitive outcomes examined. These findings suggest that higher levels of physical activity and perceived social support, together with lower levels of loneliness, are associated with better cognitive functioning in older adults (Table 2).

### 3.3. Multiple Linear Regression Analysis for Global Cognitive Function

A multiple linear regression analysis was conducted to examine the independent associations of perceived social support, loneliness, and physical activity with global cognitive performance, as assessed by the MoCA, after adjusting for age, sex, and educational level. The overall model was statistically significant (F(6,105) = 12.376, *p* < 0.001), explaining 41.4% of the variance in MoCA scores (R^2^ = 0.414; adjusted R^2^ = 0.381). Higher levels of perceived social support were independently associated with better global cognitive performance (β = 0.229, *p* = 0.006). Likewise, greater physical activity was positively associated with MoCA scores (β = 0.342, *p* < 0.001). In contrast, higher levels of loneliness showed a nominal association with poorer cognitive performance (β = −0.190, *p* = 0.022); however, this association did not remain statistically significant after FDR correction. Older age was also independently associated with lower MoCA scores (β = −0.310, *p* < 0.001). However, sex (β = −0.061, *p* = 0.432) and educational level (β = −0.063, *p* = 0.411) were not significantly associated with global cognitive performance (Table 3). Collinearity diagnostics indicated no evidence of problematic multicollinearity, with variance inflation factor (VIF) values ranging from 1.03 to 1.20.

### 3.4. Multiple Linear Regression Analysis for Verbal Fluency

A multiple linear regression analysis was conducted to examine the independent associations of perceived social support, loneliness, and physical activity with verbal fluency performance (ISAAC), adjusting for age, sex, and educational level. The overall model was statistically significant (F(6,105) = 2.603, *p* = 0.022), explaining 12.9% of the variance in ISAAC scores (R^2^ = 0.129; adjusted R^2^ = 0.080). Physical activity showed a nominal, positive association with verbal fluency performance (β = 0.214, *p* = 0.031), indicating that participants reporting higher levels of physical activity achieved better scores on the ISAAC test; however, this association did not remain statistically significant after FDR correction. In contrast, perceived social support (β = 0.138, *p* = 0.170), loneliness (β = −0.095, *p* = 0.339), age (β = −0.089, *p* = 0.337), sex (β = −0.032, *p* = 0.731), and educational level (β = −0.121, *p* = 0.193) were not significantly associated with ISAAC scores after adjustment (Table 4). Collinearity diagnostics indicated no evidence of problematic multicollinearity, with variance inflation factors ranging from 1.03 to 1.20.

### 3.5. Linear Regression Analysis for Processing Speed, Visual Search, and Executive Function

A multiple linear regression model was conducted to examine the independent associations of perceived social support, loneliness, and physical activity with processing speed and visual search performance, as assessed by the TMT-A, adjusting for age, sex, and educational level. The overall model was statistically significant (F(6,105) = 7.690, *p* < 0.001), explaining 30.5% of the variance in TMT-A completion time (R^2^ = 0.305; adjusted R^2^ = 0.266). Among the predictors included in the model, perceived social support was significantly associated with TMT-A performance (β = −0.253, *p* = 0.005). Higher levels of perceived social support were associated with shorter completion times, indicating better processing speed and visual search performance. Physical activity was also significantly associated with TMT-A performance (β = −0.302, *p* = 0.001), with higher levels of physical activity being associated with shorter completion times. In addition, older age was independently associated with longer completion times (β = 0.166, *p* = 0.048), indicating poorer performance. Loneliness was not statistically significantly associated with TMT-A performance (β = 0.163, *p* = 0.069). Sex (β = 0.065, *p* = 0.440) and educational level (β = 0.024, *p* = 0.774) were not significantly associated with TMT-A performance after adjustment (Table 5). Collinearity diagnostics indicated no evidence of problematic multicollinearity, with variance inflation factors ranging from 1.03 to 1.20.

Independent associations of perceived social support, loneliness, and physical activity with executive function, assessed by the TMT-B, were examined after adjusting for age, sex, and educational level. The overall model was statistically significant (F(6,105) = 13.385, *p* < 0.001), explaining 43.3% of the variance in TMT-B completion time (R^2^ = 0.433; adjusted R^2^ = 0.401). Among the predictors included in the model, perceived social support was significantly associated with TMT-B performance (β = −0.263, *p* = 0.001). Higher levels of perceived social support were associated with shorter completion times, indicating better executive functioning. Loneliness showed a nominal, positive association with TMT-B completion time (β = 0.189, *p* = 0.020), suggesting that greater loneliness was related to poorer executive performance; however, this association did not remain statistically significant after FDR correction. Physical activity was also independently associated with TMT-B performance (β = −0.350, *p* < 0.001), with higher levels of physical activity associated with shorter completion times. In addition, older age was independently associated with longer completion times (β = 0.285, *p* < 0.001), indicating poorer executive functioning. Sex (β = 0.054, *p* = 0.481) and educational level (β = 0.025, *p* = 0.736) were not significantly associated with TMT-B performance after adjustment (Table 6). Collinearity diagnostics indicated no evidence of problematic multicollinearity, with variance inflation factors ranging from 1.03 to 1.20.

### 3.6. Linear Regression Analysis for Processing Speed

A multiple linear regression model was conducted to examine the independent associations of perceived social support, loneliness, and physical activity with processing speed as measured by the DSST, adjusting for age, sex, and educational level. The overall model was statistically significant (F(6,105) = 5.251, *p* < 0.001), explaining 23.1% of the variance in DSST performance (R^2^ = 0.231; adjusted R^2^ = 0.187). Among the predictors included in the model, physical activity was positively associated with DSST performance (β = 0.280, *p* = 0.003), indicating that participants reporting higher levels of physical activity achieved better processing speed scores. Age was negatively associated with DSST performance (β = −0.213, *p* = 0.016), suggesting that older participants tended to perform less well on the task. Perceived social support (β = 0.151, *p* = 0.110) and loneliness (β = −0.149, *p* = 0.114) were not significantly associated with DSST performance after adjustment. Likewise, sex (β = −0.062, *p* = 0.484) and educational level (β = −0.052, *p* = 0.549) were not significant predictors (Table 7). Collinearity diagnostics indicated no evidence of problematic multicollinearity, with variance inflation factors ranging from 1.03 to 1.20.

### 3.7. Linear Regression Analysis for Attention and Concentration

A multiple linear regression model was conducted to examine the independent associations of perceived social support, loneliness, and physical activity with attention and concentration performance (D2_TOTAL), adjusting for age, sex, and educational level. The overall model was statistically significant, F(6,105) = 9.543, *p* < 0.001, explaining 35.3% of the variance in D2_TOTAL scores (R^2^ = 0.353; adjusted R^2^ = 0.316). Among the predictors included in the model, perceived social support showed a nominal positive association with attention and concentration performance (β = 0.194, *p* = 0.026), indicating that individuals reporting greater social support achieved better scores on the d2 test; however, this association did not remain statistically significant after FDR correction. Physical activity was also positively associated with D2_TOTAL scores (β = 0.357, *p* < 0.001), suggesting that higher levels of physical activity were related to better attentional performance. Age was negatively associated with D2_TOTAL scores (β = −0.266, *p* = 0.001), indicating poorer attentional performance among older participants. Loneliness was not statistically significantly associated with d2 performance (β = −0.162, *p* = 0.062). In contrast, sex (β = −0.080, *p* = 0.334) and educational level (β = −0.055, *p* = 0.525) were not significantly associated with D2_TOTAL performance after adjustment (Table 8). Collinearity diagnostics indicated no evidence of problematic multicollinearity, with variance inflation factors ranging from 1.03 to 1.20.

### 3.8. Interaction Analyses

Additional hierarchical multiple regression analyses were conducted to examine whether the associations of perceived social support, loneliness, and physical activity with cognitive performance varied as a function of their interactions. The addition of the three interaction terms significantly improved model fit for global cognitive performance (MoCA; ΔR^2^ = 0.054, *p* = 0.020) and TMT-B performance (ΔR^2^ = 0.059, *p* = 0.010). In contrast, the interaction block did not significantly increase the explained variance for verbal fluency (Isaac Set Test; ΔR^2^ = 0.041, *p* = 0.176), TMT-A performance (ΔR^2^ = 0.034, *p* = 0.164), processing speed (DSST; ΔR^2^ = 0.041, *p* = 0.135), or selective attention and concentration (d2; ΔR^2^ = 0.029, *p* = 0.192). At the individual interaction-term level, nominal associations (*p* < 0.05) were observed for loneliness × physical activity with MoCA (β = 0.152, *p* = 0.048), perceived social support × loneliness with TMT-B (β = −0.163, *p* = 0.041), and loneliness × physical activity with TMT-B (β = −0.166, *p* = 0.028). However, none of the 18 individual interaction terms remained statistically significant after Benjamini–Hochberg FDR correction (all q ≥ 0.190). Therefore, these interaction findings should be interpreted as exploratory. The interaction results across all cognitive outcomes are summarized in Table 9, with FDR-adjusted results provided in Supplementary Table S1.

Simple-slope analyses indicated that greater loneliness was significantly associated with lower MoCA scores at low levels of physical activity (−1 SD; B = −0.055, SE = 0.021, *p* = 0.009, 95% CI [−0.097, −0.014]). At the mean level of physical activity, the association was weaker and did not reach statistical significance (B = −0.032, SE = 0.017, *p* = 0.057, 95% CI [−0.065, 0.001]), whereas at high levels of physical activity (+1 SD), loneliness was not associated with MoCA scores (B = −0.009, SE = 0.020, *p* = 0.655, 95% CI [−0.048, 0.030]). Thus, the negative association between loneliness and global cognitive performance was attenuated at higher levels of physical activity.

A similar pattern was observed for TMT-B. Greater loneliness was significantly associated with longer TMT-B completion times at low levels of physical activity (−1 SD; B = 3.715, SE = 1.329, *p* = 0.006, 95% CI [1.079, 6.352]). At the mean level of physical activity, this association was attenuated and did not reach statistical significance (B = 2.065, SE = 1.056, *p* = 0.053, 95% CI [−0.030, 4.161]), whereas at high levels of physical activity (+1 SD), loneliness was not associated with TMT-B performance (B = 0.415, SE = 1.247, *p* = 0.740, 95% CI [−2.058, 2.888]). These findings indicate that the association between greater loneliness and poorer TMT-B performance was evident at low, but not at average or high, levels of physical activity.

Finally, simple-slope analyses of the perceived social support × loneliness interaction showed that greater loneliness was significantly associated with longer TMT-B completion times at low levels of perceived social support (−1 SD; B = 3.802, SE = 1.211, *p* = 0.002, 95% CI [1.399, 6.205]). At the mean level of perceived social support, the association was attenuated and did not reach statistical significance (B = 2.065, SE = 1.056, *p* = 0.053, 95% CI [−0.030, 4.161]), whereas at high levels of perceived social support (+1 SD), loneliness was not associated with TMT-B performance (B = 0.329, SE = 1.476, *p* = 0.824, 95% CI [−2.599, 3.257]). Thus, the association between greater loneliness and poorer TMT-B performance was evident at low, but not at average or high, levels of perceived social support.

## 4. Discussion

The present study aimed to examine the relationships between perceived social support, loneliness, physical activity, and cognitive functioning in older adults across multiple cognitive domains, including global cognition, verbal fluency, attention, processing speed, and executive functioning. Overall, the findings indicate that psychosocial and lifestyle factors are associated with cognitive performance in later life, although the strength and consistency of these associations differed across cognitive domains after correction for multiple comparisons. Physical activity showed the broadest pattern of independent associations across cognitive outcomes. After FDR correction, its associations remained statistically significant for global cognition, visual attention and processing speed, executive function, processing speed, and selective attention and concentration, whereas its nominal association with verbal fluency did not remain significant. Perceived social support remained independently associated with global cognition, visual attention and processing speed, and executive function after FDR correction, whereas its nominal association with selective attention and concentration did not remain statistically significant. Although loneliness showed nominal independent associations with global cognition and executive function in the multivariable models, neither association remained statistically significant after FDR correction. Furthermore, although the addition of the interaction terms improved model fit for MoCA and TMT-B, none of the individual interaction terms remained significant after FDR correction. Accordingly, the interaction and subsequent simple-slope findings should be regarded as exploratory rather than as evidence of robust moderating effects. Taken together, these findings highlight physical activity as the factor showing the broadest pattern of independent associations with cognitive performance in this sample, while the findings for perceived social support were more domain-specific and those for loneliness and the interaction analyses require more cautious interpretation.

In our study, perceived social support showed positive associations with several cognitive outcomes. After FDR correction, perceived social support remained independently associated with global cognition (MoCA), visual attention and processing speed (TMT-A), and executive function (TMT-B), whereas its nominal association with selective attention and concentration (d2) did not remain statistically significant. These findings are consistent with previous systematic reviews reporting associations between greater social support and better cognitive functioning in middle-aged and older adults [38,39]. These findings suggest that the relationship between perceived social support and cognitive performance may be more evident for global cognition and executive-related processes, although the cross-sectional nature of the study precludes conclusions regarding directionality or protective effects. The interaction analyses provided additional exploratory findings. Although the perceived social support × loneliness interaction for TMT-B reached nominal significance and simple-slope analyses suggested that the association between loneliness and poorer executive performance was more evident at lower levels of perceived social support, this interaction did not remain statistically significant after FDR correction. Therefore, these findings should be considered hypothesis-generating and should not be interpreted as evidence of a robust moderating or buffering effect of perceived social support.

Our findings also align with studies that have shown associations between social participation, social networks, and improved cognitive functioning in older adults. Recent reviews have reported that participation in formal and informal social activities is linked to better cognitive performance and a lower likelihood of cognitive decline, particularly in executive functions and global cognition [40,41]. However, the literature is not entirely consistent. Some longitudinal studies have observed clear cross-sectional associations between social support and cognition, but weaker or inconsistent longitudinal relationships [42,43]. In this context, our results provide further evidence by showing independent associations between perceived social support and multiple cognitive domains, even after simultaneously controlling for loneliness, physical activity, and sociodemographic factors.

Loneliness showed an inverse relationship with virtually all cognitive measures in bivariate analyses, a finding consistent with previous research linking loneliness and social isolation to poorer cognitive performance and a higher risk of cognitive decline and dementia [44,45]. Proposed mechanisms include increased physiological stress response, neuroendocrine alterations, chronic inflammation, and reduced cognitive stimulation resulting from decreased social interaction [46]. This relationship should also be considered within the broader context of social health, as loneliness and social isolation are related but distinct constructs that may show overlapping associations with cognitive functioning in later life [47,48]. In the multivariable analyses, loneliness showed nominal independent associations with global cognition and executive function as measured by the TMT-B; however, neither association remained statistically significant after FDR correction. Therefore, these findings should be interpreted cautiously and do not provide robust evidence of an independent association between loneliness and these cognitive outcomes after accounting for multiple comparisons. The exploratory interaction analyses suggested that the association between loneliness and cognition may vary according to physical activity and perceived social support. Specifically, simple-slope analyses indicated stronger associations between loneliness and poorer MoCA and TMT-B performance at lower levels of physical activity and between loneliness and poorer TMT-B performance at lower levels of perceived social support. However, none of the corresponding interaction terms remained statistically significant after FDR correction. Accordingly, these patterns should be regarded as exploratory and hypothesis-generating rather than as evidence that physical activity or perceived social support robustly moderates the relationship between loneliness and cognitive performance. Longitudinal and adequately powered studies are needed to determine whether these conditional associations can be replicated.

The most consistent pattern of associations observed in this study involved physical activity. Physical activity was significantly correlated with all cognitive measures assessed, with these bivariate associations remaining significant after FDR correction. In the multivariable analyses, physical activity also showed the broadest pattern of independent associations across cognitive outcomes. After FDR correction, its associations remained statistically significant for global cognition (MoCA), visual attention and processing speed (TMT-A), executive function (TMT-B), processing speed (DSST), and selective attention and concentration (d2), whereas the nominal association with verbal fluency did not remain statistically significant. These findings are consistent with extensive studies identifying physical activity as an important modifiable factor associated with cognitive function during aging [49]. Although recent reviews have indicated that the effect size of physical activity on cognitive impairment may be relatively small at the population level, even modest associations may have clinical relevance given the high prevalence of cognitive impairment and the modifiable nature of physical activity [50,51]. Within the present cross-sectional sample, physical activity therefore exhibited the broadest and most consistent pattern of independent associations among the factors examined, although these findings should not be interpreted as demonstrating a causal or protective effect. Exploratory interaction and simple-slope analyses suggested that associations between loneliness and MoCA and TMT-B performance may differ according to physical activity level. However, because the corresponding individual interaction terms did not remain statistically significant after FDR correction, these findings should be regarded as hypothesis-generating and require confirmation in longitudinal and adequately powered studies.

Age also showed nominal associations with most of the cognitive domains assessed in the multivariable models. Older age was associated with worse scores on the MoCA, DSST, and d2, as well as with longer completion times on the TMT-A and TMT-B. These findings are consistent with the established evidence on cognitive aging, characterized by age-related declines in processing speed, attention, and executive functions. In contrast, age was not significantly associated with verbal fluency in the present study.

This study has several strengths. First, it simultaneously assessed multiple cognitive domains using instruments widely used in research and clinical practice. Second, it considered psychosocial and behavioral factors together within the same analytical models, allowing for the estimation of their independent contributions. Third, the additional interaction analyses allowed us to examine whether the associations between psychosocial factors and cognition varied according to physical activity and perceived social support, revealing patterns that were not apparent from the main-effects models alone. Finally, the analysis of different cognitive domains made it possible to identify specific patterns that would have gone unnoticed using only a global measure of cognition. However, some limitations must be acknowledged. The cross-sectional design prevents the establishment of causal relationships and does not allow for determining the temporal direction of the observed associations. This limitation is particularly relevant to the interaction findings; although higher physical activity and perceived social support were associated with an attenuation of the relationship between loneliness and some cognitive outcomes, these results should not be interpreted as demonstrating protective or buffering effects. Furthermore, the measures of social support, loneliness, and physical activity were obtained through self-reporting, and therefore may be subject to recall bias or social desirability bias. This limitation may be particularly relevant to physical activity assessment because the IPAQ requires participants to recall the frequency and duration of activities performed during the previous seven days. Given that the present sample consisted of older adults with MCI, cognitive difficulties may have affected the accuracy of activity recall and introduced measurement bias. Consequently, associations involving physical activity should be interpreted with caution. Future studies should complement self-reported physical activity with objective measures, such as accelerometry or wearable activity-monitoring devices, to obtain more accurate estimates of physical activity in older adults with cognitive impairment. The interaction analyses also involved multiple cognitive outcomes and interaction terms; therefore, these findings should be considered exploratory and interpreted cautiously pending replication in larger samples. Future longitudinal studies should explore the mechanisms that link these factors to the trajectory of cognitive aging and determine whether physical activity and social support prospectively modify the relationship between loneliness and cognitive decline.

## 5. Conclusions

The present study provides evidence that psychosocial and lifestyle factors are associated with cognitive functioning in older adults, although the strength and consistency of these associations vary across cognitive domains. Among the variables examined, physical activity emerged as the most consistent correlate of cognitive performance and was independently associated with global cognition, attention, processing speed, and executive functioning after adjustment for age, sex, educational level, perceived social support, and loneliness. Perceived social support was also independently associated with selected cognitive outcomes, particularly global cognition, visual attention and processing speed, and executive functioning, whereas the associations observed for loneliness were less consistent after adjustment for covariates and correction for multiple comparisons. Overall, these findings suggest that higher levels of physical activity and perceived social support are associated with more favorable cognitive performance in older adults. However, given the cross-sectional design, these associations should not be interpreted as causal or protective effects. Longitudinal and interventional studies are needed to establish their directionality and to determine whether targeting physical activity, social support, and loneliness may contribute to the preservation of cognitive health and the prevention of cognitive decline in older adults.

## Figures and Tables

**Table 1:** Sociodemographic, clinical, and psychosocial characteristics of the sample.

Sociodemographic Variables (*n* = 112)		
Age, years		71.04 ± 3.31
Sex, *n* (%)	Male	41 (36.6%)
	Female	71 (63.4%)
Occupational status, *n* (%)	Retired	81(72.3%)
	Employed	0 (0.0%)
	Unemployed	31 (27.7%)
Marital status, *n* (%)	Single	20 (17.9%)
	Married	61 (54.5%)
	Divorced, separated, widowed	31 (27.7%)
Educational status, *n* (%)	No formal education	19 (17.0%)
	Primary education	61 (54.5%)
	Secondary education	24 (21.4%)
	University education	8 (7.1%)
Weight, kg		72.88 ± 12.68
Height, m		1.67 ± 0.13
BMI, kg/m^2^		26.41 ± 4.91
Cognitive characteristics	MoCA score, mean ± SD	21.71 ± 1.19
	MoCA score, range	20–25
	Isaac Set Test, mean ± SD	26.90 ± 2.52
	TMT-A, s, median [IQR]	109 [67]
	TMT-B, s, median [IQR]	163 [138]
	DSST, mean ± SD	46.82 ± 6.09
	d2 total, mean ± SD	159.46 ± 18.05
Psychosocial characteristics	MSPSS total, mean ± SD	5.31 ± 0.45
	UCLA Loneliness Scale, mean ± SD	49.38 ± 5.82
Physical activity	IPAQ, MET-min/week, mean ± SD	2033.75 ± 639.93

Values are presented as mean ± standard deviation or median [interquartile range], as appropriate, for quantitative variables and as frequency (percentage) for categorical variables.

**Table 2:** Correlations between psychosocial factors, physical activity, and cognitive outcomes (*n* = 112).

Variable	MoCA	Isaac	TMT-A	TMT-B	DSST	d2
Family Support	0.304 **	0.252 **	−0.333 **	−0.334 **	0.247 **	0.301 **
Friends Support	0.325 **	0.099	−0.258 **	−0.342 **	0.226 *	0.283 **
Significant Others Support	0.290 **	0.225 *	−0.316 **	−0.326 **	0.218 *	0.245 **
MSPSS Total	0.413 **	0.234 *	−0.404 **	−0.449 **	0.294 **	0.368 **
Loneliness	−0.358 **	−0.198 *	0.312 **	0.365 **	−0.270 **	−0.314 **
IPAQ	0.428 **	0.262 **	−0.400 **	−0.452 **	0.339 **	0.428 **

Values are Pearson correlation coefficients (r). * *p* < 0.05; ** *p* < 0.01. Given the non-normal distributions of TMT-A and TMT-B completion times, sensitivity analyses using Spearman’s rank correlation coefficients yielded comparable results and did not alter the interpretation of the associations. The prespecified FDR correction was applied only to the 18 correlations involving the MSPSS total score, loneliness, and physical activity across the six cognitive outcomes; FDR-adjusted q-values for these correlations are provided in Supplementary Table S1.

**Table 3:** Multiple linear regression model examining the association of psychosocial factors and physical activity with global cognitive function (MoCA).

Predictor	B	SE	β	95% CI	*p*-Value
Total MSPSS	0.603	0.215	0.229	0.176 to 1.029	0.006
Loneliness	−0.039	0.017	−0.190	−0.072 to −0.006	0.022
IPAQ	0.001	0.000	0.342	0.000 to 0.001	<0.001
Age	−0.111	0.027	−0.310	−0.166 to −0.057	<0.001
Sex	−0.150	0.190	−0.061	−0.526 to 0.227	0.432
Educational level	−0.089	0.108	−0.063	−0.304 to 0.125	0.411

Model statistics: R = 0.644; R^2^ = 0.414; Adjusted R^2^ = 0.381; F(6,105) = 12.376; *p* < 0.001. MSPSS, Multidimensional Scale of Perceived Social Support; IPAQ, International Physical Activity Questionnaire; SE, standard error; CI, confidence interval. The *p*-values shown are unadjusted. FDR-adjusted q-values are provided in Supplementary Table S1.

**Table 4:** Multiple linear regression model examining the association of psychosocial factors and physical activity with verbal fluency (Isaac Test) (*n* = 112).

Predictor	B	SE	β	95% CI	*p*-Value
Total MSPSS	0.766	0.555	0.138	−0.334 to 1.867	0.170
Loneliness	−0.041	0.043	−0.095	−0.127 to 0.044	0.339
IPAQ	0.001	0.000	0.214	0.000 to 0.002	0.031
Age	−0.068	0.070	−0.089	−0.208 to 0.072	0.337
Sex	−0.169	0.490	−0.032	−1.140 to 0.802	0.731
Educational level	−0.366	0.279	−0.121	−0.920 to 0.188	0.193

Model statistics: R = 0.360; R^2^ = 0.129; Adjusted R^2^ = 0.080; F(6,105) = 2.603; *p* = 0.022. MSPSS, Multidimensional Scale of Perceived Social Support; IPAQ, International Physical Activity Questionnaire; SE, standard error; CI, confidence interval; ISAAC = Set Test of Verbal Fluency. The *p*-values shown are unadjusted. FDR-adjusted q-values are provided in Supplementary Table S1.

**Table 5:** Multiple linear regression model examining the association of psychosocial factors and physical activity with processing speed and visual search (TMT-A) (*n* = 112).

Predictor	B	SE	β	95% CI	*p*-Value
Total MSPSS	−28.001	9.831	−0.253	−47.494 to −8.508	0.005
Loneliness	1.402	0.762	0.163	−0.109 to 2.912	0.069
IPAQ	−0.024	0.007	−0.302	−0.037 to −0.010	0.001
Age	2.502	1.248	0.166	0.028 to 4.977	0.048
Sex	6.727	8.678	0.065	−10.480 to 23.933	0.440
Educational level	1.425	4.950	0.024	−8.389 to 11.240	0.774

Model statistics: R = 0.553; R^2^ = 0.305; Adjusted R^2^ = 0.266; F(6,105) = 7.690; *p* < 0.001. MSPSS, Multidimensional Scale of Perceived Social Support; IPAQ, International Physical Activity Questionnaire; SE, standard error; CI, confidence interval; TMT-A, Trail Making Test Part A. The *p*-values shown are unadjusted. FDR-adjusted q-values are provided in Supplementary Table S1.

**Table 6:** Multiple linear regression model examining the association of psychosocial factors and physical activity with executive function (TMT-B) (*n* = 112).

Predictor	B	SE	β	95% CI	*p*-Value
Total MSPSS	−45.270	13.810	−0.263	−72.652 to −17.888	0.001
Loneliness	2.524	1.070	0.189	0.403 to 4.646	0.020
IPAQ	−0.043	0.010	−0.350	−0.061 to −0.024	<0.001
Age	6.702	1.753	0.285	3.226 to 10.178	<0.001
Sex	8.627	12.190	0.054	−15.543 to 32.797	0.481
Educational level	2.354	6.953	0.025	−11.433 to 16.140	0.736

Model statistics: R = 0.658; R^2^ = 0.433; Adjusted R^2^ = 0.401; F(6,105) = 13.385; *p* < 0.001. MSPSS, Multidimensional Scale of Perceived Social Support; IPAQ, International Physical Activity Questionnaire; SE, standard error; CI, confidence interval; TMT-B, Trail Making Test Part B. The *p*-values shown are unadjusted. FDR-adjusted q-values are provided in Supplementary Table S1.

**Table 7:** Multiple linear regression model examining the association of psychosocial factors and physical activity with DSST (*n* = 112).

Predictor	B	SE	β	95% CI	*p*-Value
Total MSPSS	2.272	1.404	0.151	−0.511 to 5.055	0.110
Loneliness	−0.309	0.193	−0.149	−0.691 to 0.074	0.114
IPAQ	0.003	0.001	0.280	0.001 to 0.005	0.003
Age	−0.391	0.160	−0.213	−0.709 to −0.074	0.016
Sex	−0.827	1.177	−0.062	−3.160 to 1.506	0.484
Educational level	−0.368	0.612	−0.052	−1.582 to 0.846	0.549

Model statistics: R = 0.480; R^2^ = 0.231; Adjusted R^2^ = 0.187; F(6,105) = 5.251; *p* < 0.001. MSPSS, Multidimensional Scale of Perceived Social Support; IPAQ, International Physical Activity Questionnaire; SE, standard error; CI, confidence interval; DSST, Digit Symbol Substitution Test. The *p*-values shown are unadjusted. FDR-adjusted q-values are provided in Supplementary Table S1.

**Table 8:** Multiple linear regression model examining the association of psychosocial factors and physical activity with selective attention and concentration (d2) (*n* = 112).

Predictor	B	SE	β	95% CI	*p*-Value
Total MSPSS	7.726	3.426	0.194	0.932 to 14.520	0.026
Loneliness	−0.501	0.265	−0.162	−1.026 to 0.025	0.062
IPAQ	0.010	0.002	0.357	0.005 to 0.014	<0.001
Age	−1.450	0.435	−0.266	−2.312 to −0.587	0.001
Sex	−3.587	3.697	−0.080	−10.917 to 3.742	0.334
Educational level	−1.219	1.912	−0.055	−5.011 to 2.574	0.525

Model statistics: R = 0.594; R^2^ = 0.353; Adjusted R^2^ = 0.316; F(6,105) = 9.543; *p* < 0.001. MSPSS = Multidimensional Scale of Perceived Social Support; IPAQ = International Physical Activity Questionnaire; SE = standard error; CI = confidence interval. The *p*-values shown are unadjusted. FDR-adjusted q-values are provided in Supplementary Table S1.

**Table 9:** Hierarchical regression analyses examining interaction effects among perceived social support, loneliness, and physical activity across cognitive outcomes.

Outcome	R^2^ Main-Effects Model	R^2^ Interaction Model	ΔR^2^	*p* for ΔR^2^	Social Support × Loneliness β (*p*)	Social Support × Physical Activity β (*p*)	Loneliness × Physical Activity β (*p*)
MoCA	0.414	0.468	0.054	**0.020**	0.138 (0.090)	−0.048 (0.564)	0.152 (0.048)
Isaac	0.129	0.170	0.041	0.176	0.163 (0.110)	0.203 (0.053)	0.017 (0.857)
TMT-A	0.305	0.339	0.034	0.164	−0.177 (0.053)	0.041 (0.648)	0.083 (0.354)
TMT-B	0.433	0.493	0.059	**0.010**	−0.163 (0.041)	0.010 (0.905)	−0.166 (0.028)
DSST	0.231	0.271	0.041	0.135	0.125 (0.175)	−0.059 (0.511)	0.162 (0.072)
d2	0.353	0.382	0.029	0.192	0.118 (0.178)	0.071 (0.427)	0.131 (0.112)

Note. Main-effects models included age, sex, educational level, perceived social support, loneliness, and physical activity. Interaction models additionally included perceived social support × loneliness, perceived social support × physical activity, and loneliness × physical activity. Continuous predictors were mean-centered before computing interaction terms. Values for interaction terms are standardized regression coefficients (β) with unadjusted *p*-values in parentheses. Nominally significant interaction terms (*p* < 0.05) are shown in bold; however, none of the 18 individual interaction terms remained statistically significant after Benjamini–Hochberg FDR correction (all q ≥ 0.190). FDR-adjusted *p*-values are reported in Supplementary Table S1.

## Data Availability

The data presented in this study are available upon request from the corresponding author. The data are not publicly available because, due to the sensitive nature of the questions asked in this study, participants were assured raw data would remain confidential and would not be shared.

## Supplementary Materials

The following supporting information can be downloaded at https://www.mdpi.com/article/10.3390/jcm15187207/s1, Table S1: Unadjusted *p*-values and Benjamini–Hochberg false discovery rate (FDR)-adjusted q-values for correlation analyses, main-effect regression coefficients, and interaction terms.

## Abbreviations

The following abbreviations are used in this manuscript:

CI	Confidence Interval
d2	d2 Attention Test
DSST	Digit Symbol Substitution Test
IPAQ	International Physical Activity Questionnaire
ISAAC	Isaac Set Test of Verbal Fluency
MoCA	Montreal Cognitive Assessment
MSPSS	Multidimensional Scale of Perceived Social Support
SE	Standard Error
TMT-A	Trail Making Test Part A
TMT-B	Trail Making Test Part B
VIF	Variance Inflation Factor
β	Standardized Regression Coefficient
B	Unstandardized Regression Coefficient
R^2^	Coefficient of Determination

## References

[1] Gianfredi V., Nucci D., Pennisi F., Maggi S., Veronese N., Soysal P. (2025). Aging, longevity, and healthy aging: The public health approach. Aging Clin. Exp. Res..

[2] Callaway J., Strozza C., Christensen K., Doblhammer G., Rau R., Søgaard J. (2025). Ageing populations: New challenges in longevity. BMC Public Health.

[3] Vega J.N., Newhouse P.A. (2014). Mild cognitive impairment: Diagnosis, longitudinal course, and emerging treatments. Curr. Psychiatry Rep..

[4] Niu B., Chen D., Niu Z., Wang J. (2025). Differential dementia risks of motoric cognitive risk syndrome and mild cognitive impairment among older adults in China: A 7-year cohort study. BMC Geriatr..

[5] Wang T., He H., Shi Y., Su N., Zhu M., Yan F., Liu Y., Li J., Tang M., Chen W. (2025). Prevalence, incidence and modifiable factors for subtypes of mild cognitive impairment: Results from the Longitudinal Ageing Study in China. Gen. Psychiatry.

[6] Colita E., Mateescu V.O., Olaru D.G., Popa-Wagner A. (2024). Cognitive decline in ageing and disease: Risk factors, genetics and treatments. Curr. Health Sci. J..

[7] Guarnera J., Yuen E., Macpherson H. (2023). The impact of loneliness and social isolation on cognitive aging: A narrative review. J. Alzheimers Dis. Rep..

[8] Amin S.M., Khedr M.A., Tawfik A.F., Gamal Noaman Malek M., El-Ashry A.M. (2025). The mediating and moderating role of social support on the relationship between psychological well-being and burdensomeness among elderly with chronic illness: Community nursing perspective. BMC Nurs..

[9] Krnjajić M., Mudri Ž., Barišić M., Barać I., Vujanić J., Čebohin M., Lovrić R., Major Poljak K., Farčić N. (2026). Cognitive functioning in rural older adults: The mediating role of perceived social support. Healthcare.

[10] Melendez J.C., Venegas L.C., de la Fuente C.P. (2026). Cognitive reserve and its relationship with memory changes: An analysis of the Survey of Health, Aging, and Retirement in Europe (SHARE). Int. J. Geriatr. Psychiatry.

[11] Liang A., Watt E., Gomaa N. (2025). Biological mechanisms linking social adversity and cognition. Public Health Rev..

[12] Arana-Nombera H.A., Matallana-Sánchez M.J., Castro-Suarez S., Paredes-Manrique C., Zegarra-Valdivia J.A. (2025). Impact of loneliness and social isolation on the cognition of healthy adults: A systematic review. Rev. Neuropsiquiatr..

[13] Hajek A., König H.H. (2025). Prevalence of loneliness and social isolation among individuals with mild cognitive impairment or dementia: Systematic review and meta-analysis. BJPsych Open.

[14] Lin Q., Dadar M., Oliver M., Barnes L.L., Morrison C. (2025). Exploring the influence of loneliness on cognitive decline: Differential effects in Black and White older adults. J. Gerontol. Ser. B Psychol. Sci. Soc. Sci..

[15] Finley A.J., Schaefer S.M. (2022). Affective neuroscience of loneliness: Potential mechanisms underlying the association between perceived social isolation, health, and well-being. J. Psychiatry Brain Sci..

[16] Ren Y., Savadlou A., Park S., Siska P., Epp J.R., Sargin D. (2023). The impact of loneliness and social isolation on the development of cognitive decline and Alzheimer’s disease. Front. Neuroendocrinol..

[17] Quigley A., MacKay-Lyons M., Eskes G. (2020). Effects of exercise on cognitive performance in older adults: A narrative review of the evidence, possible biological mechanisms, and recommendations for exercise prescription. J. Aging Res..

[18] Ben Ezzdine L., Dhahbi W., Dergaa I., Ceylan H.I., Guelmami N., Ben Saad H., Chamari K., Stefanica V., El Omri A. (2025). Physical activity and neuroplasticity in neurodegenerative disorders: A comprehensive review of exercise interventions, cognitive training, and AI applications. Front. Neurosci..

[19] Gaspar-Silva F., Trigo D., Magalhaes J. (2023). Ageing in the brain: Mechanisms and rejuvenating strategies. Cell. Mol. Life Sci..

[20] Nuzum H., Stickel A., Corona M., Zeller M., Melrose R.J., Wilkins S.S. (2020). Potential benefits of physical activity in MCI and dementia. Behav. Neurol..

[21] Hoffmann C.M., Petrov M.E., Lee R.E. (2021). Aerobic physical activity to improve memory and executive function in sedentary adults without cognitive impairment: A systematic review and meta-analysis. Prev. Med. Rep..

[22] Decaix T., Bonnin C., Götze K., François V., Petit C., Rivière C., Greffard S., Cognat E., Hugon J., Paquet C. (2025). Benefits of physical activity on cognitive function in patients with neurocognitive disorders: A systematic review. J. Frailty Aging.

[23] Eshkoor S.A., Hamid T.A., Mun C.Y., Ng C.K. (2015). Mild cognitive impairment and its management in older people. Clin. Interv. Aging.

[24] Knopman D.S., Petersen R.C. (2014). Mild cognitive impairment and mild dementia: A clinical perspective. Mayo Clin. Proc..

[25] Foley J.A., Foltynie T., Zrinzo L., Hyam J.A., Limousin P., Cipolotti L. (2017). Apathy and reduced speed of processing underlie decline in verbal fluency following DBS. Behav. Neurol..

[26] Jiang F., Dong Q., Jayakody D.M.P., Chen X., Mueller C., Wu B., Underwood B.R., Yan L.L., Li X., Bruck C. (2026). Hearing aid effectiveness and probable dementia risk across 33 countries: A pooled analysis of seven cohorts. Cell Rep. Med..

[27] Guo X., Fan C., Ren J., Zhang Z., Ni C., Liu Y. (2026). Multidomain intervention for dementia prevention: A scoping review. Front. Neurol..

[28] Wilson R.S., Bennett D.A. (2017). How does psychosocial behavior contribute to cognitive health in old age?. Brain Sci..

[29] Petersen R.C. (2004). Mild cognitive impairment as a diagnostic entity. J. Intern. Med..

[30] Nasreddine Z.S., Phillips N.A., Bédirian V., Charbonneau S., Whitehead V., Collin I., Cummings J.L., Chertkow H. (2005). The Montreal Cognitive Assessment, MoCA: A brief screening tool for mild cognitive impairment. J. Am. Geriatr. Soc..

[31] Isaacs B., Kennie A.T. (1973). The Set Test as an aid to the detection of dementia in old people. Br. J. Psychiatry.

[32] Reitan R.M. (1956). Trail Making Test: Manual for Administration, Scoring and Interpretation.

[33] Jaeger J. (2018). Digit Symbol Substitution Test: The case for sensitivity over specificity in neuropsychological testing. J. Clin. Psychopharmacol..

[34] Brickenkamp R., Seisdedos Cubero N. (2002). d2. Test de Atención.

[35] Zimet G.D., Dahlem N.W., Zimet S.G., Farley G.K. (1988). The Multidimensional Scale of Perceived Social Support. J. Pers. Assess..

[36] Russell D.W. (1996). UCLA Loneliness Scale (Version 3): Reliability, validity, and factor structure. J. Pers. Assess..

[37] Craig C.L., Marshall A.L., Sjöström M., Bauman A.E., Booth M.L., Ainsworth B.E., Pratt M., Ekelund U.L.F., Yngve A., Sallis J.F. (2003). International physical activity questionnaire: 12-country reliability and validity. Med. Sci. Sports Exerc..

[38] Costa-Cordella S., Arevalo-Romero C., Parada F.J., Rossi A. (2021). Social support and cognition: A systematic review. Front. Psychol..

[39] Mogic L., Rutter E.C., Tyas S.L., Maxwell C.J., O’Connell M.E., Oremus M. (2023). Functional social support and cognitive function in middle- and older-aged adults: A systematic review of cross-sectional and cohort studies. Syst. Rev..

[40] Cunha C., Voss G., Andrade R., Delerue-Matos A. (2024). Is formal social participation associated with cognitive function in middle-aged and older adults? A systematic review with meta-analysis of longitudinal studies. Behav. Sci..

[41] Cunha C., Rodrigues P., Voss G., Martinez-Pecino R., Delerue-Matos A. (2024). Association between formal social participation and cognitive function in middle-aged and older adults: A longitudinal study using SHARE data. Aging Neuropsychol. Cogn..

[42] Joyce J., Ryan J., Owen A., Hu J., McHugh Power J., Shah R., Woods R., Storey E., Britt C., Freak-Poli R. (2022). Social isolation, social support, and loneliness and their relationship with cognitive health and dementia. Int. J. Geriatr. Psychiatry.

[43] Li J., Shi L., Jiang N., Wang L., Wang Y., Zheng Z. (2026). Associations between social relationships and cognitive impairment in older adults: A systematic review of risk and protective factors. Medicine.

[44] Yin J., Lassale C., Steptoe A., Cadar D. (2019). Exploring the bidirectional associations between loneliness and cognitive functioning over 10 years: The English Longitudinal Study of Ageing. Int. J. Epidemiol..

[45] Zhang W., Zhang J., Gao N. (2025). Social isolation and cognitive decline in older adults: A longitudinal study across 24 countries. BMC Geriatr..

[46] Girotti M., Bulin S.E., Carreno F.R. (2024). Effects of chronic stress on cognitive function: From neurobiology to intervention. Neurobiol. Stress.

[47] Luchetti M., Aschwanden D., Stephan Y., Karakose S., Milad E., Miller A.A., Zavala D., Hajek A., Terracciano A., Sutin A.R. (2025). Loneliness and subjective cognitive concerns in daily life. Aging Ment. Health.

[48] Evans I.E.M., Martyr A., Collins R., Brayne C., Clare L. (2019). Social isolation and cognitive function in later life: A systematic review and meta-analysis. J. Alzheimers Dis..

[49] Iso-Markku P., Aaltonen S., Kujala U.M., Halme H.-L., Phipps D., Knittle K., Vuoksimaa E., Waller K. (2024). Physical activity and cognitive decline among older adults: A systematic review and meta-analysis. JAMA Netw. Open.

[50] Lu C.W., Ng T.C., Cheng Y.C., Su C.H. (2026). Exercise interventions for cognitive and functional outcomes in dementia: A systematic review and meta-analysis exploring dose metrics, heterogeneity, and implementation-relevant factors. Healthcare.

[51] Martín-Rodríguez A., Dalamitros A.A., Madrigal-Cerezo R., Sánchez-Conde P., Clemente Suárez V.J., Tornero Aguilera J.F. (2025). Move to Remember: The role of physical activity and exercise in preserving and enhancing cognitive function in aging—A narrative review. Geriatrics.

