# Supplementary material for: Associations of Social Support, Loneliness, and Physical Activity with Cognitive Function in Older Adults with Mild Cognitive Impairment
Source: J Clin Med. 2026 Sep 17;15(18):7207. doi: 10.3390/jcm15187207 (PMC13607460; doi:10.3390/jcm15187207)
Supplement: Supplementary file 1 [file jcm-15-07207-s001.zip › jcm-4482643-supplementary.pdf]

Supplementary Table S1. Benjamini–Hochberg false discovery rate correction for correlations, main-effect regression coefficients, and interaction terms.

Benjamini–Hochberg false discovery rate (FDR) correction was applied separately to three prespecified families of tests: correlations (18 tests), main-effect regression coefficients (36 tests), and interaction terms (18 tests). FDR-adjusted p-values (q-values) < 0.05 were considered statistically significant.

**A. Correlations**				
**Cognitive outcome**	**Predictor**	**Raw p**	**FDR-adjusted p (q)**	**Significant after FDR**
MoCA	MSPSS	0.000006	0.000036	Yes
MoCA	Loneliness	0.000106	0.000212	Yes
MoCA	IPAQ	0.000003	0.000027	Yes
Isaac	MSPSS	0.013054	0.013822	Yes
Isaac	Loneliness	0.036003	0.036003	Yes
Isaac	IPAQ	0.005306	0.005969	Yes
TMT-A	MSPSS	0.000010	0.000043	Yes
TMT-A	Loneliness	0.000828	0.001146	Yes
TMT-A	IPAQ	0.000012	0.000043	Yes
TMT-B	MSPSS	0.000676	0.001106	Yes
TMT-B	Loneliness	0.000076	0.000171	Yes
TMT-B	IPAQ	0.000057	0.000170	Yes
DSST	MSPSS	0.001638	0.002106	Yes
DSST	Loneliness	0.004032	0.004838	Yes
DSST	IPAQ	0.000253	0.000455	Yes
d2	MSPSS	0.000066	0.000170	Yes
d2	Loneliness	0.000752	0.001128	Yes
d2	IPAQ	0.000003	0.000027	Yes
**B. Main-effect regression coefficients**				
**Cognitive outcome**	**Predictor**	**Raw p**	**FDR-adjusted p (q)**	**Significant after FDR**
MoCA	Age	0.000087	0.000783	Yes
MoCA	Sex	0.432165	0.546186	No
MoCA	Education	0.411425	0.546186	No
MoCA	MSPSS	0.006070	0.019865	Yes
MoCA	Loneliness	0.021866	0.056227	No
MoCA	IPAQ	0.000044	0.000576	Yes
Isaac	Age	0.337154	0.469559	No
Isaac	Sex	0.730582	0.756644	No
Isaac	Education	0.193143	0.302311	No
Isaac	MSPSS	0.170052	0.278267	No
Isaac	Loneliness	0.339126	0.469559	No
Isaac	IPAQ	0.030702	0.069080	No
TMT-A	Age	0.047520	0.100631	No
TMT-A	Sex	0.439983	0.546186	No
TMT-A	Education	0.773937	0.773937	No
TMT-A	MSPSS	0.005290	0.019044	Yes
TMT-A	Loneliness	0.068549	0.129882	No
TMT-A	IPAQ	0.000777	0.004662	Yes
TMT-B	Age	0.000224	0.001613	Yes

TMT-B	Sex	0.480676	0.561938	No
TMT-B	Education	0.735626	0.756644	No
TMT-B	MSPSS	0.001417	0.006377	Yes
TMT-B	Loneliness	0.020153	0.055808	No
TMT-B	IPAQ	0.000022	0.000576	Yes
DSST	Age	0.016234	0.048702	Yes
DSST	Sex	0.483891	0.561938	No
DSST	Education	0.549115	0.599035	No
DSST	MSPSS	0.110145	0.194638	No
DSST	Loneliness	0.113539	0.194638	No
DSST	IPAQ	0.002863	0.011452	Yes
d2	Age	0.001189	0.006115	Yes
d2	Sex	0.333597	0.469559	No
d2	Education	0.525310	0.590974	No
d2	MSPSS	0.026281	0.063074	No
d2	Loneliness	0.061823	0.123646	No
d2	IPAQ	0.000048	0.000576	Yes
**C. Interaction terms**				
**Cognitive outcome**	**Interaction**	**Raw p**	**FDR-adjusted p (q)**	**Significant after FDR**
MoCA	MSPSS × Loneliness	0.089768	0.223630	No
MoCA	MSPSS × IPAQ	0.564343	0.677212	No
MoCA	Loneliness × IPAQ	0.047838	0.190487	No
Isaac	MSPSS × Loneliness	0.109832	0.223630	No
Isaac	MSPSS × IPAQ	0.052913	0.190487	No
Isaac	Loneliness × IPAQ	0.856661	0.904825	No
TMT-A	MSPSS × Loneliness	0.052607	0.190487	No
TMT-A	MSPSS × IPAQ	0.648151	0.729170	No
TMT-A	Loneliness × IPAQ	0.354465	0.531697	No
TMT-B	MSPSS × Loneliness	0.041481	0.190487	No
TMT-B	MSPSS × IPAQ	0.904825	0.904825	No
TMT-B	Loneliness × IPAQ	0.027585	0.190487	No
DSST	MSPSS × Loneliness	0.174542	0.291322	No
DSST	MSPSS × IPAQ	0.510851	0.656808	No
DSST	Loneliness × IPAQ	0.071814	0.215442	No
d2	MSPSS × Loneliness	0.178030	0.291322	No
d2	MSPSS × IPAQ	0.427114	0.591389	No
d2	Loneliness × IPAQ	0.111815	0.223630	No

Note: Raw *p*-values correspond to the original unadjusted analyses. The Benjamini–Hochberg false discovery rate (FDR) procedure was applied separately to three prespecified families of tests: (A) correlations (18 tests), (B) main-effect regression coefficients (36 tests), and (C) interaction terms (18 tests). FDR-adjusted *p*-values (*q*-values) < 0.05 were considered statistically significant. “Yes” indicates statistical significance after FDR correction ($q < 0.05$), whereas “No” indicates that the result did not meet the FDR-adjusted significance threshold. MSPSS, Multidimensional Scale of Perceived Social Support; IPAQ, International Physical Activity Questionnaire; MoCA, Montreal Cognitive Assessment; TMT-A, Trail Making Test Part A; TMT-B, Trail Making Test Part B; DSST, Digit Symbol Substitution Test.
